# Tracheal Replacement Therapy with a Stem Cell‐Seeded Graft: Lessons from Compassionate Use Application of a GMP‐Compliant Tissue‐Engineered Medicine

**DOI:** 10.1002/sctm.16-0443

**Published:** 2017-05-24

**Authors:** Martin J. Elliott, Colin R. Butler, Aikaterini Varanou‐Jenkins, Leanne Partington, Carla Carvalho, Edward Samuel, Claire Crowley, Peggy Lange, Nicholas J. Hamilton, Robert E. Hynds, Tahera Ansari, Paul Sibbons, Anja Fierens, Claire McLaren, Derek Roebuck, Colin Wallis, Nagarajan Muthialu, Richard Hewitt, David Crabbe, Sam M. Janes, Paolo De Coppi, Mark W. Lowdell, Martin A. Birchall

**Affiliations:** ^1^ Tracheal Team Great Ormond Street Children's Hospital London United Kingdom; ^2^ Lungs for Living Research Centre UCL Respiratory, University College London United Kingdom; ^3^ Centre for Cell Gene & Tissue Therapeutics, Royal Free Hospital & UCL London United Kingdom; ^4^ Department of Paediatric Surgery Great Ormond Street Children's Hospital and UCL Institute of Child Health London United Kingdom; ^5^ Department of Surgical Research Northwick Park Institute of Medical Research, Northwick Park Hospital Harrow United Kingdom; ^6^ Department of Interventional Radiology Great Ormond Street Children's Hospital and UCL Institute of Child Health London United Kingdom; ^7^ Department of Respiratory Medicine Great Ormond Street Children's Hospital and UCL Institute of Child Health London United Kingdom; ^8^ Department of Paediatric Surgery Leeds General Infirmary Leeds United Kingdom; ^9^ UCL Ear Institute and The Royal National Throat Nose and Ear Hospital London United Kingdom

**Keywords:** Trachea, Bioengineering, Tissue engineering, Bioartificial organs, Regenerative medicine, Tissue scaffolds

## Abstract

Tracheal replacement for the treatment of end‐stage airway disease remains an elusive goal. The use of tissue‐engineered tracheae in compassionate use cases suggests that such an approach is a viable option. Here, a stem cell‐seeded, decellularized tissue‐engineered tracheal graft was used on a compassionate basis for a girl with critical tracheal stenosis after conventional reconstructive techniques failed. The graft represents the first cell‐seeded tracheal graft manufactured to full good manufacturing practice (GMP) standards. We report important preclinical and clinical data from the case, which ended in the death of the recipient. Early results were encouraging, but an acute event, hypothesized to be an intrathoracic bleed, caused sudden airway obstruction 3 weeks post‐transplantation, resulting in her death. We detail the clinical events and identify areas of priority to improve future grafts. In particular, we advocate the use of stents during the first few months post‐implantation. The negative outcome of this case highlights the inherent difficulties in clinical translation where preclinical in vivo models cannot replicate complex clinical scenarios that are encountered. The practical difficulties in delivering GMP grafts underscore the need to refine protocols for phase I clinical trials. Stem Cells Translational Medicine *2017;6:1458–1464*

**Significance Statement**

We present a compassionate use case of tracheal tissue engineering using a cell‐seeded, decellularized donor trachea. The case involved application of full good manufacturing practice standards to create a manufactured advanced medical product for transplantation.

## Introduction

Congenital tracheal stenosis is rare and causes severe airway compromise due to the presence of complete tracheal rings. Surgical approaches with patch tracheoplasty augment the trachea but often fail in the long term because of re‐stenosis 1. Over the past decade, slide tracheoplasty has become the mainstay of treatment, as it significantly improves morbidity and mortality over other surgical reconstruction techniques 2, 3. However, despite these advances in reconstructive surgery, there are still children, teenagers, and adults with advanced disease that are beyond the reach of conventional therapy and for whom only palliation is possible 4. Thus, alternative strategies for these rare and, on occasion, life‐threatening cases are necessary.

Tracheal replacement therapy has been proposed as a solution for patients with severe end‐stage disease 5. In children, reconstruction with a cadaveric trachea homograft is feasible and, in short‐segment (cervical) disease, short‐term to medium‐term results have been encouraging. It is unclear if the same outcomes apply to long‐segment disease 6. Additionally, homografts have procurement difficulties and are currently unregulated. Allotransplantation also involves long‐term immunosuppression with the concomitant risks. The use of tissue‐engineered grafts based on synthetic scaffolds offers theoretical advantages, although first results have proven generally disappointing 7, 8, in part as a result of their failure to integrate with recipient tissue. Meanwhile, tissue‐engineered grafts based on decellularized scaffold constructs have remained under consideration 9, 10, 11.

Clinical applications of tissue‐engineered hollow organ constructs have been reported by a number of groups. Atala and colleagues partially reconstructed a bladder with a collagen‐polyglycolic acid scaffold seeded with urothelial and muscle cells expanded in culture from biopsy 12. Although initial reports have been encouraging, a larger clinical trial was interrupted due to a high incidence of adverse effects 13. Airway constructs based on both decellularized (“biologic”) and synthetic scaffolds have been transplanted in adults for compassionate reasons in association with respiratory epithelial cells and variably differentiated mesenchymal stromal cells (MSCs). However, protocols vary widely, products have not been manufactured as advanced medicines to good manufacturing practice (GMP) compliance (U.K.; cGMP in the U.S.), outcome reports have been limited, and some aspects of these cases are controversial 9, 14.

Initial reports suggest optimism for reconstructing airways by tissue engineering approaches, but data on long‐term outcomes are still scarce. The first adult transplanted with an autologous bone marrow MSC‐derived chondrocyte and autologous respiratory epithelial cell‐seeded decellularized scaffold has a five‐year follow‐up report 10, 14. We previously reported the four‐year follow‐up outcome of a child transplanted with an autologous cell‐seeded tissue‐engineered construct, combined with the use of exogenous growth factors at the time of the procedure [11, 15].

Here, we describe GMP methods developed to produce a decellularized tracheal scaffold seeded with autologous cells as an advanced medicine specifically for compassionate use in a teenage girl after extensive multidisciplinary team discussion. We describe the adverse clinical outcome in order to add constructively to the published literature relating to tissue‐engineered airways and use our experience of this case to provide hypotheses for future research and emphasize the need for transparent, formal clinical trials. We feel that it is essential that all such relevant cases reach the readership.

## Materials and Methods

### The Recipient

**Figure 1:**
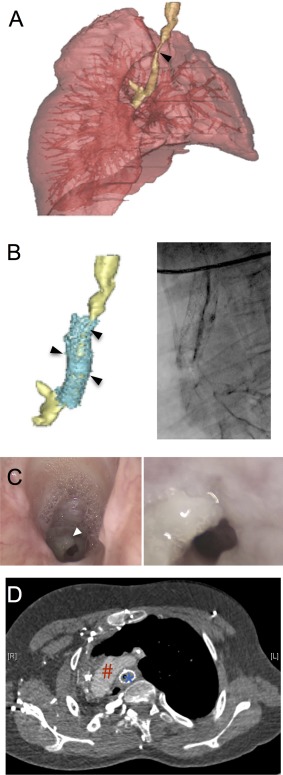
Preoperative evaluation of the airway demonstrating long‐segment tracheal stenosis with a single lung. **(A)**: Three‐dimensional (3D) model of the tracheobronchial tree demonstrating extent of stenosis (arrow) and tortuous trachea with trifurcation of the airway. **(B)**: 3D model (left) of the trachea demonstrating multiple stents (cyan) and their relation to the stenosis (arrows); corresponding bronchogram (right). **(C)**: Endoscopic photograph of the airway at the level of subglottis (left) and at the stenosis (right). White arrow indicates granulation tissue. **(D)**: Preoperative computed tomography angiogram demonstrating stenotic trachea with stent in relation to arch of aorta. Blue asterisk demonstrates stent location; red hashtag indicates arch of aorta.

A girl, born with a single left lung and long‐segment congenital tracheal stenosis (Fig. 1A), underwent a tracheoplasty with a lateral costal cartilage graft repair at 2 months of age. Following surgery, failure to extubate and ongoing malacia prompted immediate insertion of balloon‐expandable stainless steel stents (Palmaz stent, Cordis Corporation, Miami Lakes, FL, https://www.cordis.com/en_us.html; Fig. 1B). This allowed her to be extubated with mild intermittent positive pressure ventilatory support. Proactive management of stents was performed with endoscopic monitoring and balloon dilatation as required. At 4 years of age, the child underwent a pericardial patch tracheoplasty due to recurrent stenosis following failed serial balloon dilatations. Incomplete removal of the stents resulted in recurrent granulation tissue formation and re‐stenosis along the length of the repaired trachea, which was conservatively managed (Fig. 1C and 1D). Eventually, a tracheostomy was necessary, and the child became bilevel positive airway pressure (BiPAP) ventilation‐dependent at night.

At 15 years of age, she suffered a home respiratory arrest but was successfully resuscitated. Clinical deterioration, combined with the potential for further respiratory arrests, prompted clinical teams to consider tracheal replacement therapy in the absence of a satisfactory conventional reconstructive option. The child was referred to and assessed at Great Ormond Street Hospital for Children (GOSH; London, U.K.) by the multidisciplinary (“tracheal”) team, licensed by U.K. Specialist Commissioners for tracheal reconstructions in children. The multidisciplinary team was as previously described 16, with additional representation from clinicians and scientists familiar with graft and cell manufacture protocols. Consideration was given to alternate strategies, including palliation and preserved homograft replacement. Given the group's previous experience and success with autologous stem cell‐based tracheal transplant 11, 15, a tissue‐engineered construct was offered to the child and her family. The time course of her deterioration permitted procurement of a donor trachea for GMP‐compliant decellularization and concomitant expansion of autologous MSCs and epithelial cells in a fully licensed cell therapy facility (The Centre for Cell, Gene and Tissue Therapeutics, Royal Free Hospital and University College London, London, U.K.) for autologous reseeding in a bioreactor ex vivo. After further multidisciplinary discussion, approval by the Clinical Ethics Committee at GOSH and under licensing of the Human Tissue Authority (HTA) and the Medicines and Healthcare Products Regulatory Agency (MHRA, U.K. equivalent to the U.S. Food and Drug Administration [FDA]), the child, family, and clinical team agreed to the procedure with fully informed consent. The procedure took place in February 2012 at GOSH.

### Pre‐Transplant Preparation

The tracheal graft was a decellularized allogeneic trachea obtained from a cadaveric donor and seeded ex vivo with cell culture‐expanded bone marrow‐derived MSCs and nasal‐derived epithelial cells. In the European Union, scaffolds containing human cells are regulated as tissue‐engineered advanced therapeutic medicinal products (ATMPs). A major challenge was the implementation of GMP‐compliant production processes in order to satisfy regulatory requirements. This product was manufactured to GMP compliance and released for implantation as an unlicensed medicine under manufacturing authorization from the U.K. MHRA according to predefined release criteria (see supplemental online data).

The tracheal scaffold was derived from a human donor obtained through the U.K. national transplant services (National Health Services Blood and Transplant Tissue Services) under HTA licensing, and the donor was screened for potential transplant‐transmissible diseases in accordance with European regulations. The donor trachea was matched for normal tracheal diameter and procured such that length would be in excess of the host diseased segment.

The decellularization protocol applied to the trachea involved detergent enzymatic processing under vacuum pressure as previously described 17, 18 but using equivalent, GMP‐compliant decellularization reagents. Briefly, tissue was thawed to room temperature over 24 hours. Decellularization was performed under vacuum in a stainless steel Ricordi chamber with ports and a pressure gauge to measure the vacuum (1 Torr). Decellularization steps were all performed under vacuum on an orbital shaker at 140 rpm unless stated otherwise (supplemental online Fig. 4). The trachea was incubated in detergent solution containing 0.25% sodium deoxycholate, 0.25% TritonX‐100 in Hank's balanced salt solution for 24 hours at 37°C. This was followed by a wash step, which consisted of two cycles of wash solution for 2 hours at 37°C and further incubation for 44 hours at 4°C on an undulating orbital shaker at 20 rpm. The trachea was then incubated in nuclease solution at 37°C for 24 hours, followed by wash steps, as described above. The nuclease solution with wash steps was repeated once, and the scaffold product was temporarily stored in University of Wisconsin cold storage solution at 4°C prior to cell engraftment.

**Figure 2:**
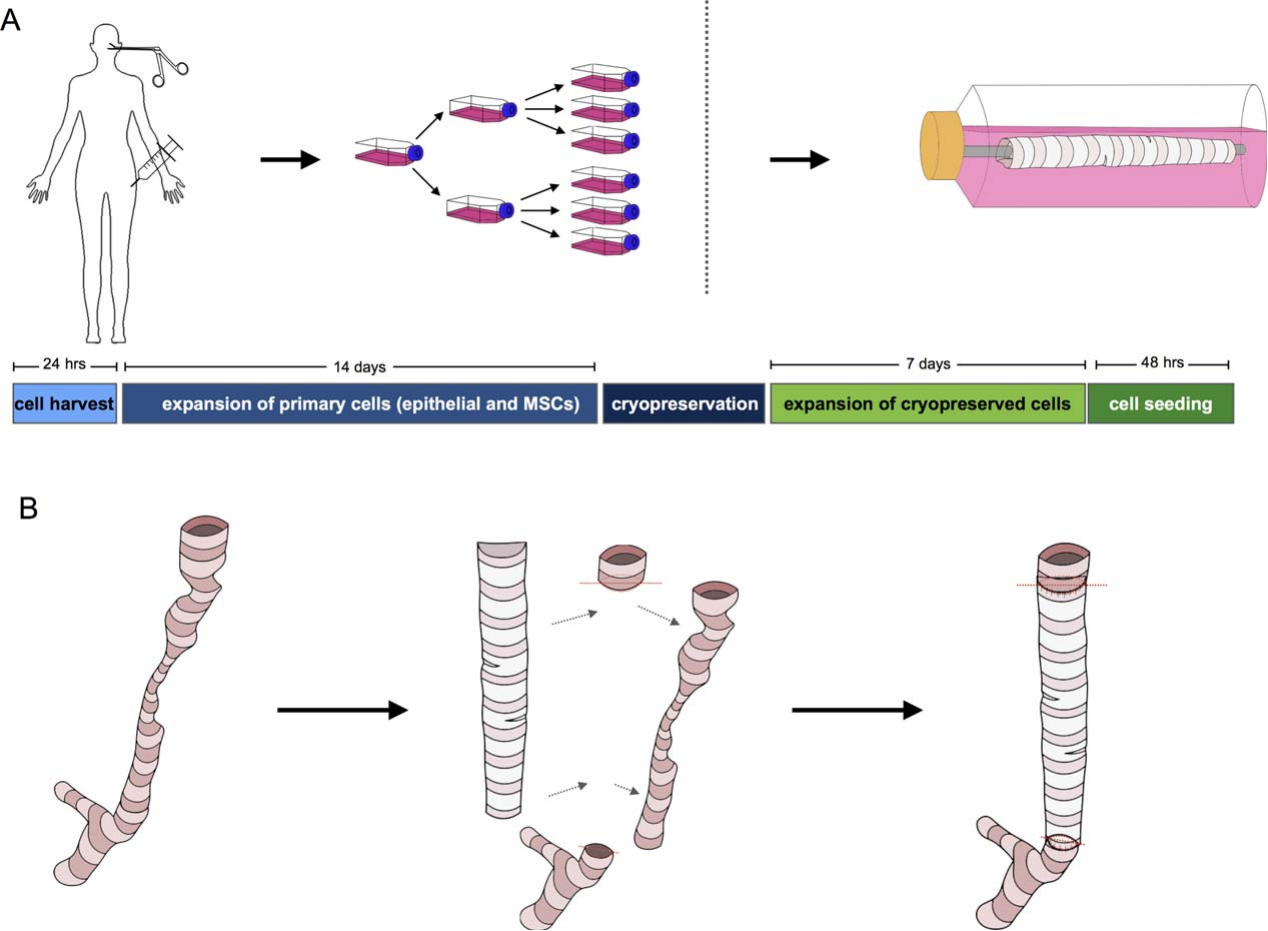
Strategy for delivering the cell‐seeded decellularized trachea scaffold. **(A)**: Donor respiratory epithelial cells were isolated from mucosal biopsies taken from the nose and expanded by explant culture. Epithelial cells were passaged over 14 days prior to cryopreservation. Bone marrow aspirate was also obtained, and MSCs were expanded in culture over the same time period before cryopreservation. Prior to transplantation, cells were thawed and expanded for 7 days, seeded onto the decellularized scaffold in a bioreactor, and incubated for 48 hours. **(B)**: Schematic representation of surgical strategy with implantation of the tracheal product in place of the diseased trachea. Abbreviation: MSCs, mesenchymal stromal cells.

Autologous MSCs were obtained from a bone marrow aspirate prior to graft production and expanded through four cell passages to obtain sufficient MSCs for both quality control and scaffold seeding (supplemental online Fig. 1). Similarly, respiratory epithelial cells were obtained from mucosal biopsies obtained from the nasal septum and inferior turbinate and expanded as explant cultures. Cells were seeded onto the scaffold in a bioreactor prior to transplantation (Fig. 2A and 2B; see supplemental online data).

### Tracheal Replacement Surgery

**Figure 3:**
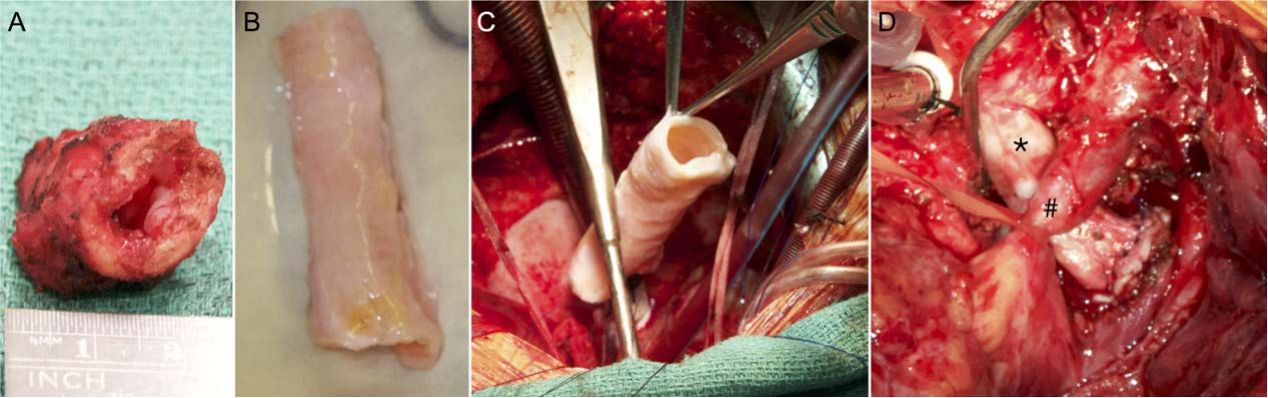
Surgical transplantation of a good manufacturing practice‐compliant tissue‐engineered tracheal product. **(A)**: Macroscopic image showing a representative segment of the resected trachea with stenosis. **(B)**: Tracheal product immediately prior to transplantation. **(C)**: Delivery of the cell‐seeded decellularized tracheal scaffold under cardiopulmonary bypass. **(D)**: Tracheal product in situ with arch of aorta crossing over the anterior surface (encircled with sling). Black asterisk indicates tracheal product. Black hashtag indicates the retracted aortic arch.

Following induction with general anesthesia, a redo sternotomy was performed. Mobilization of an adherent single left lung was required to obtain access to a right‐sided heart. Following cannulation of the aorta and inferior vena cava, hypothermic cardiopulmonary bypass was commenced with full heparinization. The trachea was densely adherent to adjacent structures, including the aorta. It was mobilized thoroughly and resected to within 1 cm of the tracheostomy and 1 cm above the carina (Fig. 3A). Partial migration of the stent through the tracheal wall meant it was not possible to fully remove the metallic stent over the posterior wall. Both proximal and distal ends of the replacement trachea (Fig. 3B and 3C) were anastomosed with interrupted horizontal mattress sutures (4/0 PDS II) and tested for an air leak prior to weaning off bypass (Fig. 3D). Laparoscopic exploration of the abdomen revealed insufficient omentum to form a “wrap” around the construct because of previous abdominal surgery, including fundoplication and gastrostomy. Pleural and mediastinal drains were inserted, and the sternum was closed in layers. The tracheostomy tube was changed to size 5.0 Portex Bivona (Smiths Medical, Ashford, U.K., https://www.smiths-medical.com). Postoperative antibiotics were given, including amikacin and teicoplanin.

## Results

**Figure 4:**
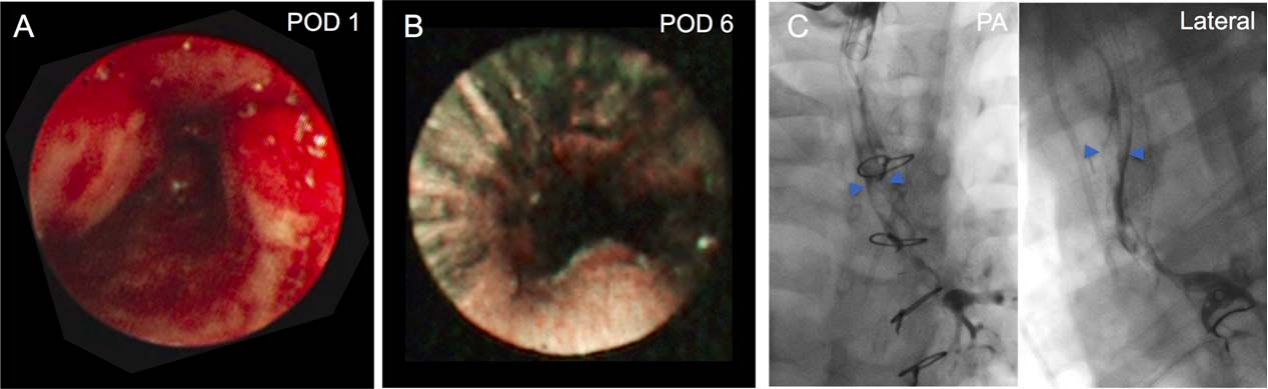
Postoperative evaluation of the airway. Endoscopic view of transplanted airway at POD 1 **(A)** and POD 6 **(B)**. **(C)**: Bronchographic evaluation at POD 7 with PA (left) and lateral (right) views. Blue arrows show contrast within the margins of the transplanted trachea. Abbreviations: PA, posteroanterior; POD, postoperative day.

Immediate postoperative bronchoscopy revealed the graft to be patent and the anastomoses intact. The posterior trachealis wall appeared to partially prolapse into the airway, but this was improved by positive airway pressure (Fig. 4A). It was possible to pass a flexible bronchoscope (size 4.0 mm; Olympus Corporation, Shinjuku, Tokyo, https://www.olympus-global.com) through the graft to the carina. Post‐surgery ventilator support was immediately provided through the tracheostomy but with minimal pressure settings. The tracheostomy was downsized within 24 hours to an uncuffed fenestrated tube.

The patient tolerated pureed foods with no signs of aspiration and spoke in full sentences within 24 hours. She continued to make good progress with decreasing ventilator support and noninvasive positive pressure ventilation. Microlaryngoscopy and bronchoscopy was used for graft monitoring and removal of excess secretions and mucus plugs. Dynamic information via bronchography showed a patent airway with only mild malacia, and so the tracheostomy was upsized to uncuffed 5.5 Bivona tube. Minimal pressure support was required with positive end expiratory pressure setting of 10/5 cmH_2_O. Humidified oxygen with regular nebulizers was provided throughout with regular chest physiotherapy. Initially, the patient mobilized without problems and walked distances greater than 100 meters, a distance not previously possible for her. Monitoring of the airway continued with both direct endoscopic evaluation and bronchography (Fig. 4B and 4C).

After 13 days of intensive care support, the patient was transferred to her local care center, where similar monitoring was performed. She was on intermittent positive airway pressure support, tolerating up to 1 hour off device at a time. Evaluation of the airway continued according to clinical need and, whilst there was a mild observed degree of malacia, no intervention was necessary. At day 15, however, the patient developed ventilatory compromise. During bronchoscopic evaluation, there had been progression of the tracheal graft narrowing. Clinicians suspected that a possible acute extrinsic compressive event had taken place (an intrathoracic hemorrhage was hypothesized). A prolonged respiratory arrest ensued, with cerebral hypoxic injury and edema. The girl's condition was considered irreversible at 24 hours, and she died following withdraw of ventilator support with parental consent.

## Discussion

Tracheal replacement is widely proposed as a solution for patients with end‐stage upper airway disease for which conventional treatment strategies currently fail 19. The complexity of upper airway disease has meant there has been no single, clear strategy for replacement. A plethora of potential tracheal scaffolds and surgical techniques for implantation and/or reconstruction have been described 20. Such solutions have included synthetic materials 8, allogeneic trachea 21, aortic grafts 22 and composite reconstruction from autologous tissue. The heterogeneous presentation of tracheal pathology has added to the complexity of the picture, and it is therefore unsurprising that individual groups have developed their own methods to treat severe airway compromise.

Tissue‐engineered grafts have been used clinically to replace urogenital tissues, including partial bladder replacement, vagina, and urethra 12, 23. Other tissue‐engineered solutions include epidermal 24, 25, corneal 26, 27, vascular 28, 29, and tracheal grafts 14, 15. Common to many of these have been the use of scaffolds that are biomimetic of native tissue and the seeding of cells normally resident at the target site. To avoid rejection, seeded cells are generally autologous, that is, derived from patient biopsies. Cells are often expanded in vitro until required numbers are achieved and checked for viability, absence of infection, and retention of the desired phenotype. Cells are subsequently seeded onto the scaffold ex vivo in a bioreactor and the construct used as a transplant graft/organ 30. Recellularization appears to be particularly critical for airway grafts, as grafts without cells uniformly fail to remodel appropriately 31. The precise role of seeded cells, particularly multipotent cells such as MSCs, is not known but hypotheses include participation in repair by repopulating the graft (e.g., differentiating into chondrocytes), modifying the regenerative process through altered host immune response (e.g., promoting pro‐repair “M2” macrophage response) 32, and accelerating angiogenesis. Targeted research in each of these areas should be a priority.

A tissue‐engineered graft was chosen for this compassionate use application due to the observed success of similar grafts used under emergent conditions for an adult 14 and a young boy 15, both of whom achieved long‐term survival. While postoperative intervention has been required in both cases in the form of ballooning and dilatation, this is not dissimilar to the aftercare required in conventional airway surgery. Both patients have patent airways and have been able to return to work and school, respectively. The methods applied have aimed to replace the airway with a “like‐for‐like” graft that is anatomically and functionally similar to native trachea and does not require immunosuppression, both of which are intuitive advantages.

With airway grafts, there has been an increasing emphasis on the importance of a viable epithelial layer to improve repair and regeneration 33, 34. In this case, we were able to deliver epithelial cells to GMP compliance for a tissue‐engineered graft. However, the delivery of autologous cells represented a significant challenge due to the difficulties in expanding sufficient numbers of cells from small samples/biopsies. Subsequent to this clinical case, we have developed a protocol for the rapid expansion of human airway epithelial cells that retain basal cell marker expression and differentiation capacity, potentially overcoming this hurdle 35.

In our previous compassionate tracheal replacement case at GOSH 36, we used an internal biodegradable stent 37. This provided insurance against a reduction in biomechanical integrity, but we observed an intense inflammatory response within the lumen that contributed to significant morbidity during the early postoperative period. This was at least in part due to the stent material, as shown by subsequent experience with stents in children 38. Also, this earlier graft was not seeded with selected and expanded epithelial cells and was instead reliant on resected mucosal biopsies secured on the luminal surface with a stent. Due to delayed re‐epithelialization, the eventual epithelial covering was thought to be derived from ingrowth of recipient epithelium rather than from the biopsies 11. In the case we describe here, time requirements permitted autologous cell expansion and seeding in a bioreactor. We opted for a non‐stented scaffold graft in order to preserve these cells as far as possible and avoid the morbidity associated with absorbable stents.

It is reasonable to hypothesize that the biomechanical rigidity of a tissue‐engineered implant in early weeks and months may reduce as remodeling and revascularization occurs, and that this process contributed to the severe obstructive event in the present case. We were unable to confirm this, as postmortem retrieval of the graft was not possible (declined by request of patient's relatives). For the same reason, we were unable to confirm whether the mechanism for acute obstruction was a primary failure of the graft, severe malacia, or secondary to extraluminal compression. Nonetheless, the clinical events lead us to recommend stenting of tracheal grafts for at least the first few months following transplantation. More evidence surrounding alterations in biomechanical performance of bioengineered tracheas in relevant in vivo models or from human trials experience is required. If biomechanical integrity is altered long term, external stenting may also represent a potential solution. It is noteworthy that previous porcine studies have not observed such mechanical changes when transplanted 31, which may represent inherent differences between species and/or the fact that in vivo models cannot replicate the pathology seen in complex airway patients.

To our knowledge, this was the first occasion in which a tissue‐engineered trachea was prepared to full clinical standards (GMP, U.K.). The constituent cells and scaffold, as well as the eventual composite seeded graft, were prepared according to validated standard operating procedures and subject to quality control and release criteria (an upper threshold for maximum DNA content after decellularization and absence of detectable HLA class I expression on decellularized scaffold; sterility of the scaffold, MSCs, and epithelial cells; absence of detectable endotoxin). As a result of this case, we have been able to identify areas for improvement in all areas of preparation and quality control, which has directly led to the protocols that are now approved by the MHRA and could inform future clinical trials of tracheal and laryngeal tissue‐engineered implants. The lessons learned are invaluable for those wishing to translate tissue‐engineered products for a range of unmet clinical needs.

There has been debate about the most scientifically valid and ethical means of introducing novel surgical technology, including those involving ATMP, as here 39. Many countries regulate the use of novel treatments for compassionate purposes. In the U.K., hospitals need specific licensing, as do the laboratories preparing the therapeutic product (a specials license). There is some variation between hospitals regarding local ethical review, but, in general, a properly constituted clinical ethics committee needs to consider the individual case and a dedicated, preferably independently monitored, consenting process developed. It is also important that patients are not approached as research subjects and that no procedures, such as biopsies and imaging, are performed that are not strictly required for clinical purposes without full discussion with and approval from the patient, their family, and appropriate ethics and regulatory bodies.

We believe reports of both success and failures of compassionate use “first‐in‐human” cases must be combined with preclinical data to build applications to regulatory bodies for clinical trials authorization (Clinical Trial Authorisation, U.K.; FDA's Investigational New Drug Application in U.S.). Conversely, failure to report interventions performed under compassionate use legislation, especially when outcomes are not favorable, may unfairly skew the literature and lead to inappropriate conclusions about safety and potential efficacy. Underreporting of negative or null outcomes is a major threat to the field and there are few incentives to prevent this type of publication bias 40, 41. We would advocate a standardized approach to form an international registry to document the application of ATMPs for compassionate use with registration, analysis, and publication of outcomes a prerequisite for granting of approval.

## Conclusion

Based on our previous successful experiences, we developed a tissue‐engineered tracheal replacement for a girl with critical tracheal stenosis unsuitable for conventional reconstructive techniques. The graft was prepared to full GMP standards. The early results were encouraging, as demonstrated by an immediate improvement in exercise tolerance. However, an acute event, hypothesized to be acute malacia and/or an intrathoracic bleed, caused airway obstruction at 3 weeks and resulted in her death. This experience has permitted us to substantially improve our GMP and clinical protocols to reduce risk and improve outcomes for future patients. Specifically, we advocate the use of indwelling stents for several months after implantation. Our GMP and clinical protocols will shape future phase I/II clinical trials of tracheal replacement. We advocate the formation of an international registry of clinically delivered ATMPs for compassionate use cases.

## Author Contributions

M.J.E.: manuscript writing, clinical care; C.R.B.: manuscript writing, clinical care; M.A.B.: manuscript writing, clinical care; M.W.L. led the GMP production of the product, critical review of manuscript; A.V.‐J.: GMP manufacture of the product; L.P.: GMP manufacture of the product; E.S.: GMP manufacture of the product; C.Carvalho: GMP manufacture of the product; C. Crowley: GMP manufacture of the product; T.A.: developed and assisted with decellularization protocols; P.L.: developed and assisted with decellularization protocols; P.S. developed and assisted with decellularization protocols; S.M.J.: led epithelial programme, critical review of manuscript; R.E.H.: manuscript writing; P.D.C.: critical review of manuscript, clinical care; N.J.H.: clinical care; A.F.: clinical care; C.M.: clinical care; D.R.: clinical care; N.M.: clinical care; R.H.: clinical care; D.C.: clinical care.

## Disclosure of Potential Conflicts of Interest

M.A.B. has research funding from joint grants with Videregen and ReNeuron. The authors indicated no potential conflicts of interest.

## Supporting information

Supporting Information
