# Supplementary material for: Tracheal Replacement Therapy with a Stem Cell‐Seeded Graft: Lessons from Compassionate Use Application of a GMP‐Compliant Tissue‐Engineered Medicine
Source: Stem Cells Transl Med. 2017 May 24;6(6):1458–64. doi: 10.1002/sctm.16-0443 (PMC5689750; doi:10.1002/sctm.16-0443)
Supplement: Supplementary file 1 — Supporting Information [file SCT3-6-1458-s001.docx]

**Supplemental Data**

**GMP MANUFACTURE OF A BIOENGINEERED TRACHEAL SCAFFOLD**

**Pre-transplant preparation**

A donor allogeneic trachea was obtained from a cadaveric donor within 48 hours post mortem. The trachea was decellularized and seeded *ex vivo* with bone marrow-derived mesenchymal stromal cells (MSCs) and nasal biopsy derived ciliated epithelial cells (ECs). Scaffolds containing human cells are regulated as tissue-engineered advanced therapeutic medicinal products (ATMPs) in the European Union (E.U.). A major challenge in this is the need to implement Good Manufacturing Practice (GMP) compliant production processes in order to satisfy regulatory requirements. This product was manufactured to GMP-compliance and released for implantation as an unlicensed medicine under manufacturing authorisation from the United Kingdom (U.K.) Medicines and Healthcare products Regulatory Agency (MHRA) according to pre-defined release criteria. In translating many of the processes to GMP, several critical reagents were not readily available as GMP-compliant but the entire manufacturing process in the decellularized trachea underwent a comprehensive risk assessment which was agreed with the chief pharmacist at each of the end-user hospitals. GMP Manufacturing of MSCs and ECs was performed in validated ‘open’ procedures in monitored Class II Microbiological Safety cabinets (grade A / ISO5) environment within a Grade B (ISO6) laboratory with in-process environmental monitoring using tryptone soya agar (TSA) settle plates (Oxoid, Basingstoke, U.K.) and continual airborne particle counting (APC; Biotest, Solihull, U.K.).

**Mesenchymal stromal cell isolation and expansion**

Under procurement licences from the U.K. Human Tissue Authority, 80-90 ml bone marrow aspirates from the iliac crest were collected into 50 ml sterile tubes (Nunc, Rokslide, Denmark) pre-loaded with 200 IU of preservative-free sodium heparin (Monoparin, Workhardt, U.K.) under local anaesthesia with informed consent. Mononuclear cells (MNCs) were obtained from the bone marrow aspirates by discontinuous density gradient centrifugation (Lymphoprep; Axis Shield Diagnostics, Dundee, U.K.) and the resulting MNC fraction counted by haematology analyser (PocH-100i; Sysmex, Milton Keynes, U.K.). MNCs were cultured in alpha-modified essential medium (α-MEM; Life Technologies, Paisley, U.K.) supplemented with 10% South American origin, European Directorate for the Quality of Medicines & HealthCare (EDQM)-approved foetal bovine serum (FBS; Lonza, Verviers, Belgium) in either T175 flasks (Nunc, Thermo Fisher Scientific, MA, U.S.A.).

Seeding densities for MSC monolayers were 5.0 x 10^4^ MNC/cm^2^ and cultures were incubated at 37°C and 5% CO_2_. After 3 days of incubation, non-adherent cells were removed, flasks were washed with Hanks Balanced Salt Solution (HBSS; Life Technologies, Paisley, U.K.) and fresh culture medium was added. Cultures were propagated by feeding every 3-5 days with supplemented α-MEM until >80% confluence was achieved. At this stage, adherent cells were trypsinized (TrypLE; Life Technologies) and re-passaged at a seeding density of 500 cells/cm^2^. Cells derived from bone marrow MNC were designated as Passage 0 and each cycle of reseeding of cells after trypsinization was considered to be one additional passage. Propagation of MSCs did not continue beyond Passage 4 or earlier if 10 population doublings was calculated to have occurred (Supplemental Table 1). MSCs were cryopreserved at each cell passage in 1.5 ml cryovials (Nunc, Thermo Fisher Scientific) or 50 ml cryocyte bags (Miltenyi Biotec, Bisley, U.K.), dependent on the cell yield, in an equal volume of cryoprotectant and cells. Cryoprotectant consisted of Human Serum Albumin (HSA; BioProducts Laboratory Ltd, Elstree, U.K.) with 20% Dimethylsulfoxide (DMSO; Wak-Chemie, Steinberg, Germany), giving a final concentration of 10% DMSO. MSCs were stored in vapour phase liquid nitrogen (LN_2_) and re-thawed when the scheduled delivery of the re-seeded tracheal graft for surgery had been confirmed. Thawed MSCs were washed in supplemented α-MEM at 200 xg for 10 minutes before passaging as described above.

Immunophenotyping of cell cultures was performed at each passage and on the MNC fraction prior to seeding according to the International Society for Cellular Therapy’s minimal criteria for definition of MSCs (Supplemental Figure 1) [1]. A minimum of 5 x 10^4^ cells were acquired after gating of viable cells using forward scatter (FSC) and side scatter (SSC) signals on a MACSQuant Analyser flow cytometer (Miltenyi Biotec) and data were analysed using FlowJo version 7.6 (TreeStar Inc, Ashland, OR, U.S.A.). Cells were adjusted to 1 x 10^6^ cells/ml in HBSS and then stained for 10 minutes at room temperature with allophycocyanin (APC)-conjugated anti-CD105, fluorescein isothiocyanate (FITC)-conjugated anti-CD90, phycoerythrin (PE)-conjugated anti-CD73, perdinin chlorophyll (PerCP)-conjugated CD45 and vioblue-conjugated anti-CD34, anti-CD14 and anti-HLA DR (all Miltenyi Biotec). Controls were appropriately isotype-matched FITC, PE and APC antibodies from the same supplier. Cell cultures were assessed every 3-5 days by phase contrast microscopy to determine cell adherence, morphology and confluence and culture medium was routinely sampled for microbiological sterility testing.

Chondrogenic differentiation of MSCs commenced from passage 1. Differentiation medium consisted of high glucose Dulbecco’s modified Eagle’s medium (DMEM; Sigma Aldrich, Saint Louis, MO, U.S.A.) supplemented with 10 ng/ml recombinant human transforming growth factor-ß3 (TGF-ß; R&D Systems, Abingdon, U.K.), 1 mM sodium pyruvate, 80 µM L-ascorbic acid 2-phosphate sesquimagnesium salt hydrate, 100 nM dexamethasone (all Sigma Aldrich), 20% dipeptiven (Fresenius Kabi, Graz, Austria) and 20% insulin-transferrin-selenium (ITS) medium. ITS medium was prepared using 20 ml sterile pharmacy grade water (Baxter, Newbury, U.K.) supplemented with 625 µg/ml insulin (Novo Nordisk, Crawley, U.K.), 625 µg/ml recombinant human transferrin, 625 ng/ml selenious acid, 535 µg/ml linoleic acid (all Sigma Aldrich), and 125 mg/ml human serum albumin (HSA; BioProducts Laboratory Ltd). Chondrogenic differentiation was induced over 48 hours in T175 culture flasks at a seeding density of 12,000 cells/cm^2^.

Microbiological testing for aerobic and anaerobic bacteria and fungi was carried out on bone marrow aspirates prior to processing and on subsequent MNC fractions after processing by inoculation of Bactec culture media bottles (Becton Dickinson, Franklin Lakes, NJ, U.S.A.). The medium from MSC cultures at all passages was also tested for microbial growth and prior to release after seeding of decellularized tracheal scaffold. No visible contaminants were detected in culture media bottles after continuous monitoring for 10 days.

| **Human Decellularized Scaffold** | | |
| --- | --- | --- |
| **Passage (P)** | **Cell Yield(x 10^6^)** | **Population Doubling** |
| P0 | 63.1 | - |
| P1 | 28.4 | 4.08 |
| P1* | 16.8 | 2.03 |
| P1** | 7.7 | - |
| P2 | 13.4 | 3.93 |
| P3 | 14.7 | 4.06 |
| **Total cells used for seeding**‡ | **24.5** |  |

**Supplemental Table 1: MSC yield and population doubling in GMP-manufactured MSC cultures.**

* Indicates MSCs that were propagated after thawing of cryopreserved cell passages.

** Indicates MSCs where chondrogenic differentiation was induced after thawing of MSCs from P0 for subsequent seeding of the decellularized tracheal scaffold.

‡ Cell number derived from P1 MSCs both differentiated and undifferentiated propagated after thawing of cryopreserved cell passages and subsequent culture.


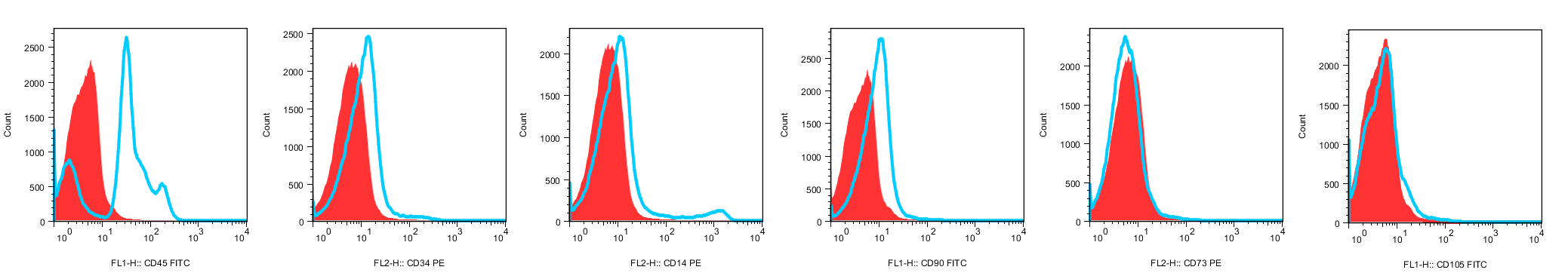

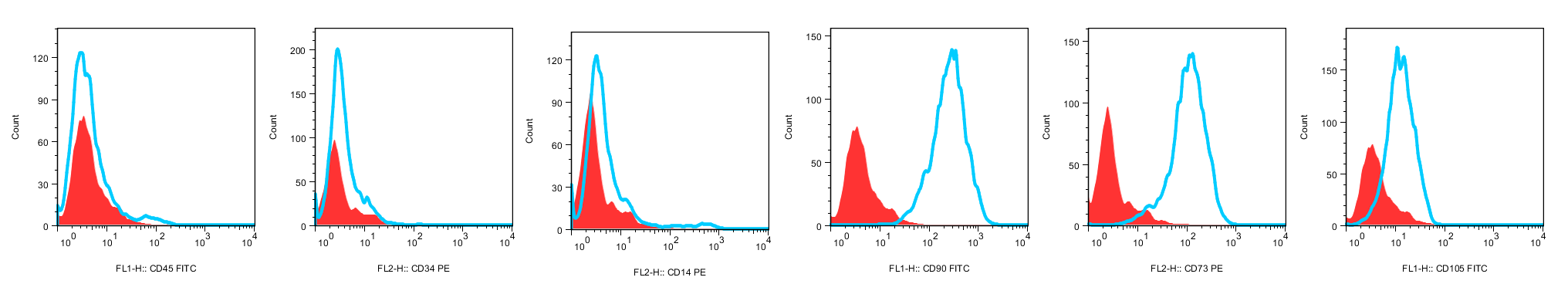

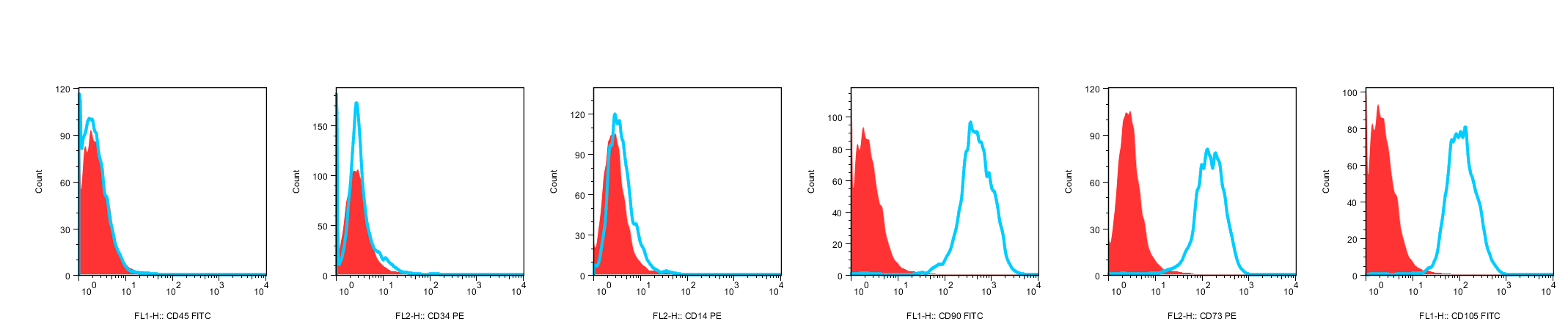


**Count**

**41.2%**

**4.1%**

**10.2%**

**5.4%**

**4.4%**

**4.5%**

**0.7%**

**3.9%**

**4.1%**

**98.7%**

**85.6%**

**94.9%**

**0.6%**

**0.9%**

**1.7%**

**99.0%**

**97.1%**

**98.5%**

**MNC – Day 0**

**P1 – Day 22**

**P2 – Day 31**

**CD45**

**CD34**

**CD14**

**CD90**

**CD73**

**CD105**

**Supplemental Figure 1: Characterization of MSCs isolated and expanded from patient bone marrow.**

Expression of CD45, CD34, CD14, CD90, CD73 and CD105 surface markers on MNC prior to culture and MSCs during passage 1 (day 22) and passage 2 (day 31). The results are presented as FACS histograms (isotype control = solid histograms; surface marker stain = histograms without fill) and are gated on cells using forward scatter versus side scatter dot plots.

**Epithelial cell isolation and expansion**

Multiple biopsies were harvested from the patient’s nasal septum and inferior turbinate and used for subsequent expansion of epithelial cells (EC). Biopsies were collected into 50 ml sterile tubes containing 150 IU of preservative free sodium heparin and 10 ml of Roswell Park Memorial Institute (RPMI) medium (Life Technologies) for each biopsy. Biopsies were dissected into tissue segments of approximately 2-5 mm in diameter and between 5 – 7 biopsies were placed into T25 culture flasks (Nunc, Thermo Fisher Scientific) and allowed to adhere before culturing in bronchial epithelial cell growth medium (BEGM; Lonza). This consisted of bronchial epithelial basal medium (BEBM; Lonza) supplemented with bovine pituitary extract, hydrocortisone, recombinant human epidermal growth factor, epinephrine, transferrin, insulin, retinoic acid, triodothyronine and gentamicin sulphate according to the manufacturer’s instructions (Lonza). Flasks were incubated for 14 days at 37°C with 5% CO_2_, with one medium change at day 7 (Supplemental Figure 2). Biopsies were re-plated in T75 culture flasks (Nunc, Thermo Fisher Scientific) with 9 – 11 biopsies per flask for continuation of EC propagation. Flasks were trypsinized (Trypsin/EDTA; Lonza) and ECs passaged at 3000-4000 cells/cm^2^ in T175 flasks in supplemented BEGM. Cell outgrowth from biopsies was designated as P0 and each subsequent re-seed after trypsinization was considered to be a cell passage. Culture of ECs was terminated at Passage 2 and biopsies were re-seeded a maximum of 3 times before discard. ECs were cryopreserved from Passage 0 to Passage 2, as previously described for MSCs. Thawed ECs, for re-seeding of scaffolds, were seeded in either T25 or T75 flasks at a density of 3500 cells/cm^2^ in supplemented BEGM. Following 5 hour incubation, a medium change was performed to remove the DMSO present from cryopreservation. Epithelial cells were >90% pure and >70% viable as defined by phase contrast light microscopy. In the same manner as microbiological testing of MSC cultures, EC culture medium was tested for aerobic and anaerobic bacteria and fungi using Bactec culture media bottles.


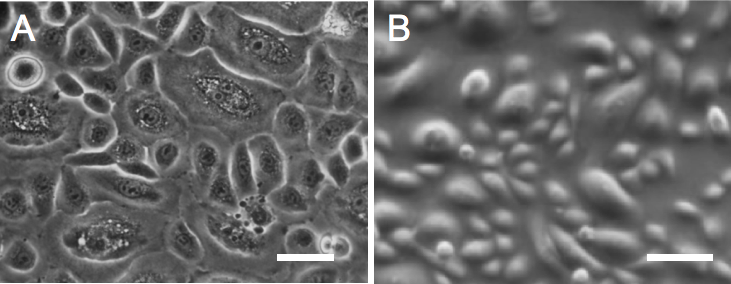


**Supplemental Figure 2: Derivation of nasal epithelial cell from patient biopsies.**

(A) Epithelial cell morphology at Passage 0 and (B) Passage 1. Scale bar = 50µm.

**Tracheal scaffold production**

We manufactured a tracheal scaffold for transplantation after seeding with bone marrow-derived MSCs and nasal biopsy-derived ECs. The allogenic tracheal scaffold was a human donor trachea supplied by NHS Blood and Transplant (NHSBT, U.K.) and decellularized using a pre-clinical validation protocol and translated to GMP compliance. Decellularization was performed in a Ricordi Chamber (Biorep Technologies Inc, Miami, FL, U.S.A.), a device which has been previously validated for clinical use in human pancreatic islet cell isolation [2].

**Decellularization of a human, allogeneic donor trachea**

An allogeneic trachea of appropriate size was retrieved in conjunction with NHSBT U.K. and transported to the Centre for Cell, Gene & Tissue Therapeutics (CCGTT), Royal Free Hospital (London, U.K.) in sterile infusion-grade University of Wisconsin (UW) solution, under a Human Tissue Authority (HTA) licence for the import, export and storage of tissue for human application. The donor tested negative after screening for statutory infectious disease markers. In GMP conditions the donor tissue was subsequently transferred into a new sterile container, suspended in fresh UW solution and frozen at -80°C.

The decelluarization procedure was developed at Northwick Park Institute for Medical Research (NPIMR) and translated to a GMP-compliant process at the CCGTT, Royal Free Hospital. The process was subjected to pre-clinical bench validation with porcine trachea and a single human trachea provided by NHSBT. [3, 4] Pre-clinical studies showed successful removal of donor cells as determined by immunohistochemical microscopy analysis and scaffolds were non-immunogenic as determined by xenogeneic implantation into rats. [3] GMP-compliant reagents were sourced with equivalence maintained with regard to composition and concentration. Decellularization was performed in a Grade A environment for ‘open’ parts of the procedure and in a Grade B environment for ‘closed’ processes. Environmental monitoring was performed using settle plates and airborne particle counting in accordance with GMP manufacturing protocols.

The trachea was removed from controlled storage at -80^o^C 13 days prior to planned surgical implantation, allowed to thaw over 24 hours before removal of the bifurcation just above the carina using a surgical scalpel (Swann Morten, Sheffield, U.K.). The trachea and bronchi were placed into a stainless steel Ricordi Chamber (Biorep) that had been sterilized by autoclaving at 131°C, a process validated by Royal Free Hospital Central Sterile Supply Department (CSSD; Supplemental Figure 4). The stainless steel Ricordi chamber was adapted with a vacuum line (Nalgene 980 braided reinforced tubing; Thermo Fisher Scientific) from the lid port and silicone tubing (Thermo Fisher Scientific) from separate medium inlet and outlet ports at the base of the chamber. An airtight Rexroth manual shut-off valve (RS Components, Corby, U.K.) was fitted into the vacuum line to ensure the Ricordi chamber could be sealed when the desired level of vacuum had been achieved. Nalgene syringe filters (Thermo Fisher Scientific, Basingstoke, UK) were fitted inline between the Ricordi chamber and the shut-off valve to maintain sterility. Decellularization was performed by depressurising the Ricordi chamber to 1 Torr (99.9% vacuum) using a vacuum pump (2F10; Telstar, Terrasa, Spain). Temperature logging (Libero Ti1; Elpro, Arundel, U.K.) of the process was performed at 5 minute intervals throughout the procedure.

The following decellularization steps were all performed under negative pressure in the Ricordi chamber on an orbital platform shaker at 140 rpm unless stated otherwise. The thawed trachea was incubated in detergent solution consisting of 0.25% sodium deoxycholate (Sigma Aldrich), 0.25% protein solubilizer x-100 (A.G. Scientific, San Diego, CA, U.S.A.) and 1% Antibiotic-Antimycotic made up in HBSS (both Life Technologies) over 24 hours at 37°C. Following the detergent step a wash step was applied. All wash steps were performed with a wash solution consisting of sterile water (Baxter, Newbury, U.K.) supplemented with 1% antibiotic-antimycotic (Life Technologies). Two cycles of wash solution for 2 hours at 37^o^C was applied followed by a further step of wash solution for 44 hours at 4^o^C on an undulating platform shaker at 20 rpm. Washing of the tracheal scaffold was followed by a 24 hour incubation with nuclease solution, consisting of 450ml perfusion medium (Lonza) supplemented with 900kU Pulmozyme (Roche, Welwyn, U.K.), 1800U of RNase (Applied Biosystems, Carlsbad, CA, U.S.A.) and 1% antibiotic-antimycotic (Life Technologies) at 37°C for 24 hours. The wash steps as described above over was applied. A further nuclease solution step with wash steps was repeated. Following this the tracheal scaffold and bronchi were aseptically removed from the Ricordi chamber and stored in 50 ml sterile tubes suspended in UW solution in preparation for cell engraftment.

Decellularized tissue from the bifurcation was used for quality control, confirming the absence of α-MHC Class I expression by immunohistochemistry and of nucleated cells by hematoxylin and eosin (H&E) staining 3 days before transplantation (Supplemental Figure 3). Sterility was confirmed by aerobic and anaerobic Bactec culture of washings collected from the final stage of the decellularization process. Following the decellularization procedure the final inner surface area of the scaffold was estimated to be 20 cm^2^.


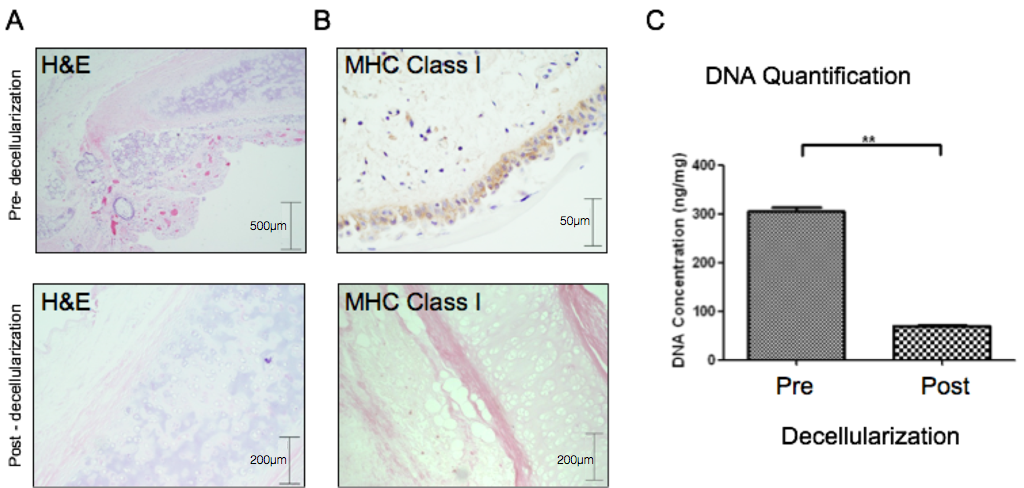


**Supplemental Figure 3: Quality control of decellularized airway by histology, immunohistochemistry and DNA quantification.**

(A) H&E staining of allogeneic, donor-derived trachea tissue showed detectable nucleated cells prior to decellularization. No nucleated cells were detectable after decellularization. Scale Bar: top panel = 500µm; bottom panel 200 µm. (B) Immunohistochemistry showed that α-MHC class I was detectable in the trachea prior to decellularization but absent after decellularization. Scale Bar: top panel = 50µm; bottom panel 200 µm. (C) Quantification of DNA showed a significant decrease in decellularized trachea compared to native tissue (** p<0.01).


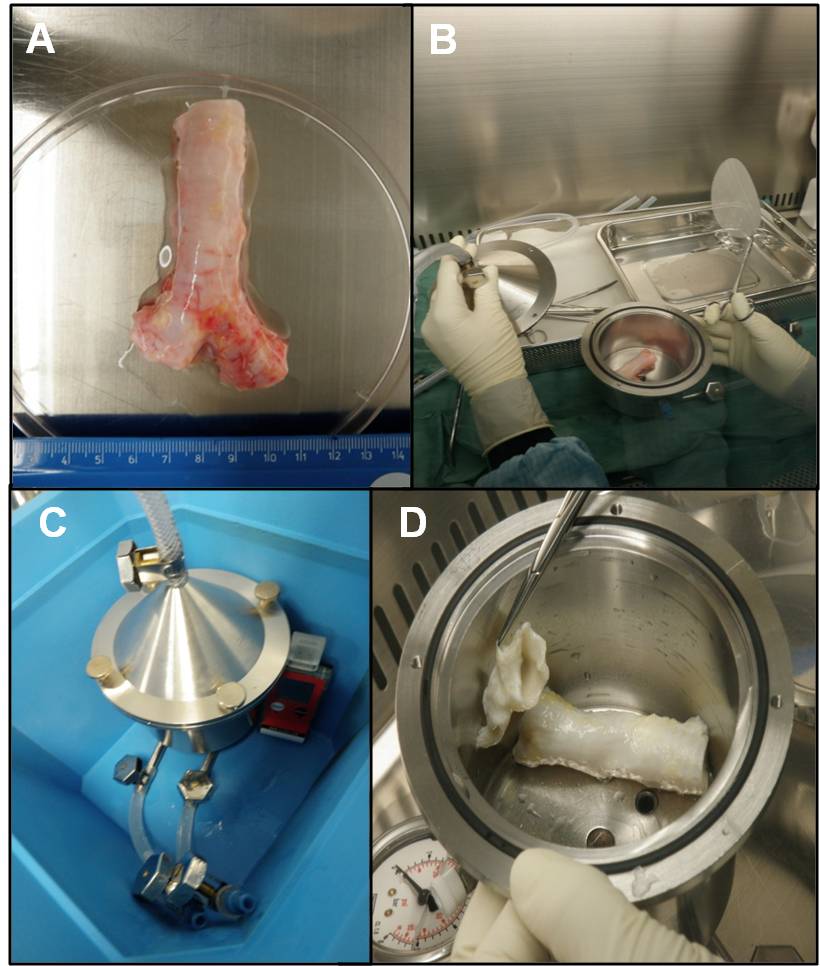


**Supplemental Figure 4: A GMP compliant vacuum-assisted decellularization process.**

(A) Pre-operative cadaveric human trachea underwent a fully GMP decellularization process in a pressurized closed-system (B, C) Ricordi chamber over 7 days before removal (D). At this point the trachea was ready for cell seeding in a bioreactor.

**GMP product manufacture**

Product manufacture and the subsequent supply of the allogeneic tracheal scaffold seeded with bone marrow-derived MSCs were generated under GMP conditions and released under an MHRA ‘specials’ licence after approval from the relevant institutional clinical ethics committees. The tracheal replacement product was approved for use as a medicine by an institutional ‘Use of Medicines Committee’ after examination of process risk assessments as previously published [5] and subsequently prescribed as an unlicensed ATMP.

**Autologous cell seeding of allogeneic trachea**

Cell seeding of a decellularized allogeneic donor trachea with bone marrow-derived MSCs, differentiated chondrocytes and nasal biopsy-derived epithelium was manufactured. A bioreactor was developed to accommodate the tracheal scaffold, consisting of a γ-irradiated disposable 2 L tissue grade roller bottle (Corning) with a custom-made polytetrafluoroethlene (PTFE) plug into which a stainless steel rod was inserted to hold the scaffold during the process of cell seeding (Supplemental Figure 5). This was carried out inside a 50 ml sterile tube with a neoprene bung (Fisher Scientific, Loughborough, U.K.) cut to size and placed on the distal end of the scaffold to allow seeding of both the luminal and outer surfaces of the scaffold. A total of 1.87 x 10^6^ ECs were re-suspended in supplemented BEGM and seeded onto the luminal surface of the tracheal scaffold by incubation of the construct at 37°C and rotation through 90° every 15 minutes until one full rotation was completed. 16.8 x 10^6^ undifferentiated MSCs were seeded onto the outer surface of the construct in supplemented α-MEM and incubated at 37°C for 1 hour prior to seeding of 7.7 x 10^6^ differentiated MSCs onto the luminal surface as previously described [6]. The tracheal construct was transferred into the bioreactor and submerged in α-MEM for 48 hours at 37°C with 5% CO_2_ before transplantation.


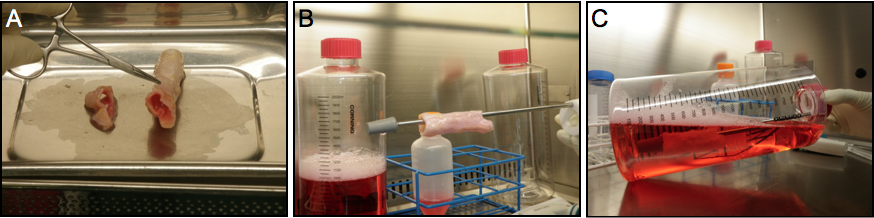


**Supplemental Figure 5: Seeding of autologous cells onto a decellularized human donor tracheal scaffold.**

(A) The trachea was removed from the bioreactor and checked visually for integrity. (B) The scaffold was assembled onto the bioreactor which consisted of a 2 L disposable tissue grade roller bottle (Corning) with a custom-made polytetrafluoroethlene (PTFE) plug into which a stainless steel rod was inserted to hold the scaffold during the cell seeding process. (C) The tracheal scaffold post-cell seeding, submerged in α-MEM before incubation at 37°C with 5% CO_2_.

**Product release criteria and transportation**

Bioengineered tracheal scaffolds seeded with bone marrow-derived MSCs and ECs derived from nasal biopsies were manufactured under an MHRA ‘specials’ licence and released as an unlicensed medicine after meeting a number of stringent criteria that confirmed the construct as ‘fit for infusion’. All human-derived tissue was procured after written donor consent and tested negative for standard transplant infectious disease markers. Release criteria required confirmation that tissue culture supernatants removed from cultures prior to cell seeding tested negative for aerobic and anaerobic bacterial and fungal contamination after a minimum of 48 hours and tested negative by Gram stain after cell seeding. Batch manufacturing records (BMRs) were completed for all processes in compliance with GMP manufacture and verified before release of the final product. Quality control of MSCs and ECs was defined as meeting acceptance based on <10 population doublings, >70% viability and >90% purity defined by phase contrast light microscopy and >90% purity of MSCs defined by surface phenotype using flow cytometry.

The custom-made bioreactor used in the manufacture of the autologous recellularized allogeneic trachea was assessed as free from visible contamination, determined by phase contrast light microscopy viewed at a magnification of x400 in accordance with the final product release criteria.

Bioengineered tracheal scaffold was released in non-supplemented α-MEM in a 50 ml sterile tube with final product labels for the named patient attached. Products were vacuum packed in labelled pouches (The Vacuum Pouch Company, Bury, U.K.) and shipped according to a validated process in a temperature controlled container (Delta T, Fernwald, Germnay) maintained at ambient temperature (8-25°C) and monitored by continuous temperature logging (Libero Ti1; Elpro, Arundel, U.K.). The product was shipped directly from the manufacturing facility to the designated operating room where its acceptance was documented on arrival according to legibility of the product container, maintenance of ambient temperature during transit and that products were intact, free from obvious contaminants and malleable.

**Supplemental References**

1. Dominici, M., et al., *Minimal criteria for defining multipotent mesenchymal stromal cells. The International Society for Cellular Therapy position statement.* Cytotherapy, 2006. **8**(4): p. 315-7.

2. Qi, M., et al., *Human pancreatic islet isolation: Part I: digestion and collection of pancreatic tissue.* J Vis Exp, 2009(27).

3. Lange, P., et al., *Pilot study of a novel vacuum-assisted method for decellularization of tracheae for clinical tissue engineering applications.* J Tissue Eng Regen Med, 2015.

4. Partington, L., et al., *Biochemical changes caused by decellularization may compromise mechanical integrity of tracheal scaffolds.* Acta Biomater, 2013. **9**(2): p. 5251-61.

5. Lowdell, M.W., M. Birchall, and A.J. Thrasher, *Use of compassionate-case ATMP in preclinical data for clinical trial applications.* Lancet, 2012. **379**(9834): p. 2341.

6. Macchiarini, P., et al., *Clinical transplantation of a tissue-engineered airway.* Lancet, 2008. **372**(9655): p. 2023-30.
